# Identification of a potent benzoxaborole drug candidate for treating cryptosporidiosis

**DOI:** 10.1038/s41467-019-10687-y

**Published:** 2019-06-27

**Authors:** Christopher S. Lunde, Erin E. Stebbins, Rajiv S. Jumani, Md Mahmudul Hasan, Peter Miller, John Barlow, Yvonne R. Freund, Pamela Berry, Rianna Stefanakis, Jiri Gut, Philip J. Rosenthal, Melissa S. Love, Case W. McNamara, Eric Easom, Jacob J. Plattner, Robert T. Jacobs, Christopher D. Huston

**Affiliations:** 1 Anacor Pharmaceuticals, Palo Alto, CA 4230 USA; 2 Department of Medicine, University of Vermont Robert R. Larner College of Medicine, Burlington, VT 05405 USA; 3 Department of Microbiology and Molecular Genetics, University of Vermont College of Agriculture and Life Sciences, Burlington, VT 05405 USA; 4 Department of Animal and Veterinary Sciences, University of Vermont College of Agriculture and Life Sciences, Burlington, VT 05405 USA; 5 Department of Medicine, University of California San Francisco, San Francisco, CA 94143 USA; 6 Calibr at Scripps Research, La Jolla, CA 92037 USA

**Keywords:** Phenotypic screening, Parasitic infection, Drug development

## Abstract

Cryptosporidiosis is a leading cause of life-threatening diarrhea in young children and causes chronic diarrhea in AIDS patients, but the only approved treatment is ineffective in malnourished children and immunocompromised people. We here use a drug repositioning strategy and identify a promising anticryptosporidial drug candidate. Screening a library of benzoxaboroles comprised of analogs to four antiprotozoal chemical scaffolds under pre-clinical development for neglected tropical diseases for *Cryptosporidium* growth inhibitors identifies the 6-carboxamide benzoxaborole AN7973. AN7973 blocks intracellular parasite development, appears to be parasiticidal, and potently inhibits the two *Cryptosporidium* species most relevant to human health, *C. parvum* and *C. hominis*. It is efficacious in murine models of both acute and established infection, and in a neonatal dairy calf model of cryptosporidiosis. AN7973 also possesses favorable safety, stability, and PK parameters, and therefore, is an exciting drug candidate for treating cryptosporidiosis.

## Abstract (web summary)

*Cryptosporidium* infection can cause severe diarrhea with limited treatment options available. Here, Lunde et al. perform a drug repositioning screen with a library of benzoxaboroles and identify AN7973 as potent inhibitor of intracellular parasite development with good efficacy in murine and neonatal dairy calf disease models.

## Introduction

Despite efforts to improve hygiene, infectious diarrhea is responsible for ∼11% of early childhood deaths^1^. A recent multicenter study of life threatening diarrhea conducted in the Indian subcontinent and Sub-Saharan Africa found that the little-studied apicomplexan parasite *Cryptosporidium*, predominantly either *Cryptosporidium parvum* or *Cryptosporidium hominis*, is amongst the two or three most important causes of childhood diarrhea^2,3^. Cryptosporidiosis is also strongly associated with child malnutrition and developmental stunting^4,5^. *Cryptosporidium* parasites were previously best known as a frequent cause of chronic diarrhea in immunocompromised people, such as those with AIDS or following organ transplantation, and in the United States they account for more than 85% of waterborne diarrheal disease for which a pathogen is identified^6–8^. Cryptosporidiosis is also a large economic concern for beef and milk producers, and infection of cattle may contribute to contamination of water supplies and human outbreaks^9,10^. In a nationwide survey of farms in the United States, *C. parvum* was present on more than 50% of dairy farms, and 48% of all calves aged 1–3 weeks were infected^9^. Despite the public health and economic impacts of *Cryptosporidium*, there is no preventive vaccine and treatment options for both humans and cattle are limited. The only approved treatment, nitazoxanide, reduces the duration of diarrhea in immunocompetent adults, but is only moderately effective in children and is equivalent to a placebo in immunocompromised patients^11–13^. Its activity in dairy calves is controversial and at best marginal^14,15^. New anti-*Cryptosporidium* drugs are urgently needed, and several promising drug leads have been reported recently^16–19^.

Benzoxaboroles are easily synthesized boron-heterocyclic compounds that have garnered interest from the pharmaceutical industry in the last decade^20^. They typically engage with proteins through interaction of an electrophilic boron atom with nucleophilic partner residues (e.g., serine, threonine or tyrosine) or with metal cations (e.g., Zn^2+^, Mg^2+^) present in the active site of enzymes, acting as a phosphate mimetic. Potent benzoxaborole inhibitors of bacterial^21,22^, fungal^23^, and protozoan^24^ pathogens have been identified that work by inhibiting various essential microbial enzymes^22,23,25–28^. Tavaborole, a leucyl-tRNA synthetase inhibitor, is approved for treatment of onychomycosis, and crisaborole, which targets human phosphodiesterase 4, was recently approved for treatment of atopic dermatitis^20,29^.

*Plasmodium* parasites, which cause malaria, are members of the phylum Apicomplexa, and are therefore genetically related to *Cryptosporidium* parasites. Given this and the recent identification of benzoxaboroles with potent antimalarial activity^28,30,31^, we screened a library of benzoxaborole compounds for anticryptosporidial activity. Here, we report identification of a novel drug candidate, AN7973, for treatment of cryptosporidiosis. AN7973 potently inhibits multiple *C. parvum* isolates and the *C. hominis* TU502 isolate in vitro, appears to be parasiticidal, has outstanding activity in rodent models of both acute and established infection, and was efficacious against *C. parvum* in a dairy calf clinical model. Its initial pharmacokinetic (PK), stability, and safety profiles are highly favorable, and indicate its potential as a new drug for treating cryptosporidiosis in all target populations.

## Results

### Drug repositioning strategy and compound screen

**Fig. 1:**
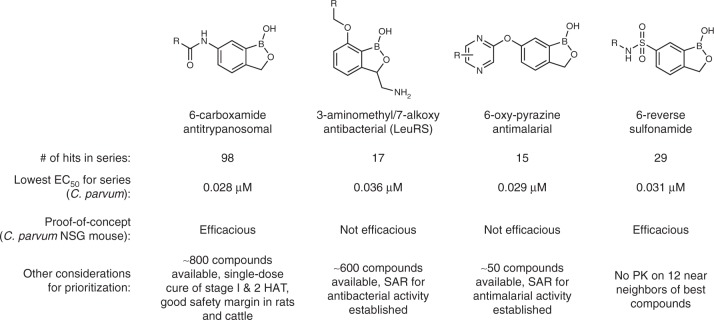
Screening results and prioritization considerations. Confirmed anticryptosporidial screening hits, lowest EC_50_, and prioritization considerations for four benzoxaborole chemical scaffolds are shown. The 6-carboxamide antitrypanosomal chemical scaffold was prioritized for further study based on a large number of available analogs, existing safety data from a human African trypanosomiasis (HAT) drug development program, and the results of a preliminary murine efficacy study

To meet the need for new anticryptosporidial drug candidates, we adopted a drug repositioning strategy to capitalize on existing benzoxaborole scaffolds and knowledge from advanced programs within Anacor’s Neglected Tropical Disease portfolio, which included antikinetoplastid programs with the Drugs for Neglected Diseases *initiative* (DND*i*) and the Global Alliance for Veterinary Medicine (GALVmed), an antimalarial program with the Medicines for Malaria Venture (MMV), and an internal antibacterial leucyl-tRNA synthetase (LeuRS) inhibitor program. A library of 7802 compounds, including analogs similar to four chemical scaffolds with antiprotozoal activity, was screened in duplicate for inhibition of *C. parvum* growth within MDCK cells using high content microscopy and adaptation of a previously described screening assay (Fig. 1)^32^. This screen yielded 403 hits with >70% inhibition at a concentration of 1 µM. Confirmatory dose-response assays were conducted in triplicate on 400 compounds available for follow-up, and an additional 222 compounds were tested to complete structure activity relationship (SAR) analyses for each scaffold. Further prioritization was then done by conducting a preliminary mouse efficacy study with one compound representative of each chemical scaffold that was selected based on availability of chemical stocks, existing potency and mouse PK data, and the potential to leverage other programs within Anacor’s Neglected Tropical Disease portfolio. AN7973 was selected for further development based on these considerations, and the remaining scaffolds were retained as alternates worthy of revisiting if necessary.

**Fig. 2:**
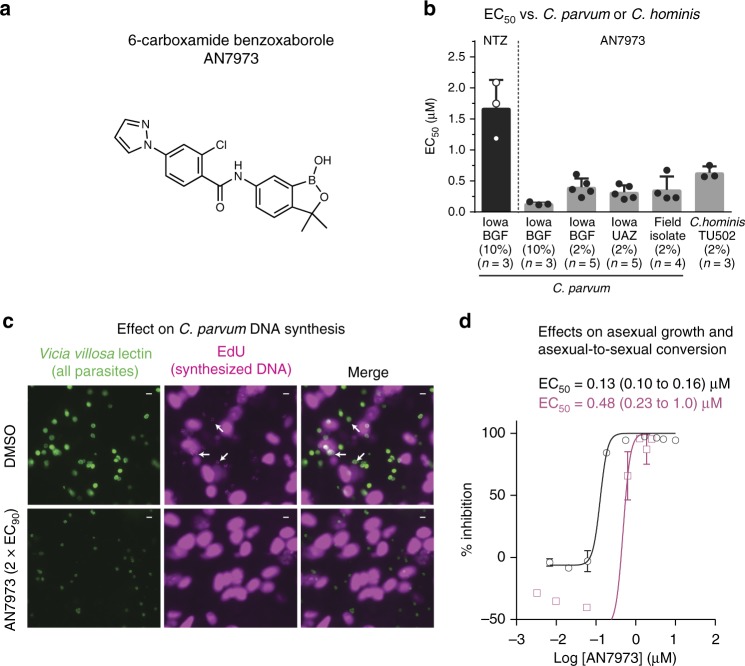
AN7973 inhibits intracellular *Cryptosporidium* replication. **a** Structure of the 6-carboxamide benzoxaborole lead AN7973. **b** In vitro efficacy of AN7973 against various *C. parvum* isolates and the *C. hominis* TU502 isolate grown within HCT-8 cells. Nitazoxanide (NTZ) is included as a positive control. BGF Bunch Grass Farms, UAZ University of Arizona. The percent fetal bovine serum present in the medium and number of replicates for each assay are given in parentheses. Data are the mean and SD. Assays for **c** through **d** were performed in 10% FBS. **c** Effect of AN7973 on intracellular DNA synthesis. *C. parvum* (Bunch Grass Farms Iowa isolate) infected host cell monolayers were incubated in the presence of the thymidine analog EdU, and AN7973 (2 × EC_90_ = 0.42 µM) or DMSO (control). All parasites are shown in green (*Vicia villosa* lectin staining) and newly synthesized DNA (incorporated EdU) is shown in cyan. Arrows indicate EdU positive parasites, which are seen only in the absence of AN7973. Representative images from 2 independent experiments are shown. Scale bars are 5 µm. **d** Potency of AN7973 against asexual growth (0–48 h (black line and circles)) and asexual-to-sexual conversion (48–72 h (cyan line and squares)) of *C. parvum* (Bunch Grass Farms Iowa isolate). Data are the means and SD for each concentration with data combined from 2 independent experiments (*n* = 4 per experiment)

AN7973 (Fig. 2a) is a benzoxaborole 6-carboxamide representative of a class of compounds active against *Trypanosoma brucei*, the cause of human African trypanosomiasis (HAT). This class of compounds is currently in human trials^33,34^. The 6-carboxamide SCYX-7158 (AN5568/acoziborole) exhibits favorable PK properties in animals and humans, e.g., prolonged exposure and half-life, and has the potential to provide a single-dose cure for HAT^35^. Related benzoxaboroles from the 6-carboxamide class have also shown good in vitro activity against human African trypanosomiasis (HAT) due to *T. br. brucei*, *T. br. gambiense*, and *T. br. rhodesiense*, and African animal trypanosomiasis (AAT) due to *T. congolense* and *T. vivax*.^36,37^. Members of this class have also shown favorable safety profiles, which enhanced the status of AN7973 for the repositioning strategy of this program.

### AN7973 selectively inhibits *Cryptosporidium* development

The structure and purity of AN7973 were determined by ^1^H NMR, ^13^C NMR, high resolution mass spectral analysis (HRMS), and high performance liquid chromatography (HPLC) (Supplementary Figs. 1 and 2).

In vitro inhibition of the Bunch Grass Farm (BGF) *C. parvum* Iowa isolate by 48 h of exposure to AN7973 in HCT-8 cells was confirmed repeatedly, with the measured EC_50_ ranging from 0.13 µM to 0.43 µM. Comparable activity was observed for inhibition of field *C. parvum* isolates, and the *C. parvum* Iowa isolate sourced from the University of Arizona Sterling Laboratory (this isolate has been propagated independently of the BGF isolate for ∼20 years). AN7973 also inhibited the *C. hominis* TU502 isolate, but with 3-fold to 4-fold higher EC_50_ (Fig. 2b). Cytotoxicity as assessed in three cell lines was not observed at concentrations up to 25 µM (i.e., the highest concentration tested (Supplementary Table 1)), yielding a selectivity index for inhibition of *Cryptosporidium* of greater than 50 in all cases.

The anticryptosporidial activity of AN7973 was further examined using a panel of in vitro assays to determine its mode of action^38^. AN7973 had no activity in an assay for *C. parvum* invasion of HCT-8 host cells (Supplementary Fig. 3). It predominantly affected intracellular *Cryptosporidium* development, since it arrested new DNA synthesis as reflected by inhibition of incorporation of the thymidine analog 5-ethynyl-2′-deoxyuridine (EdU) into newly synthesized DNA (Fig. 2c). The relative effects of AN7973 on different life cycle stages were assessed by comparing potency against asexual replication with potency in reducing the percent of parasites expressing the meiotic recombination protein DMC1, which is a biomarker for *Cryptosporidium* sexual development^38^. Unlike some compounds, e.g., the piperazine-based lead MMV665917, that have equal potency against asexual and sexual stage parasites^38^, AN7973 inhibited asexual parasite growth four times more potently than asexual-stage to sexual-stage differentiation (Fig. 2d), which was felt to be consistent with its effect on intracellular replication.

**Fig. 3:**
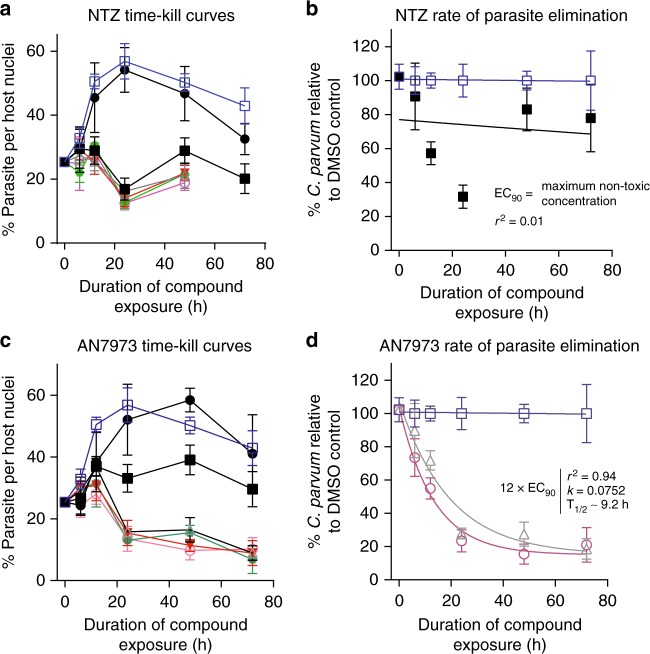
AN7973 acts rapidly to eliminate *Cryptosporidium* in vitro. Time-kill curves showing in vitro effects of nitazoxanide (NTZ) or AN7973 on *C. parvum* (Bunch Grass Farms Iowa isolate) following compound exposure. NTZ or AN7973 were added at varying concentrations ~24 h after infecting HCT-8 cell monolayers, and then parasites were stained and enumerated by high-content microscopy at the indicated time points. Different compound concentrations are indicated in all graphs as follows: open blue squares and blue lines (DMSO control); solid black circles and black lines (EC_50_); solid black squares and black lines (EC_90_); open gray triangles and gray lines (3 × EC_90_); solid red triangles and red lines (6 × EC_90_); solid green hexagons and green lines (9 × EC_90_); and open cyan circles and lines (12 × EC_90_). **a** and **c** Percentage of host cells infected vs. time for NTZ and AN7973, respectively. Data shown are the means and SD for 4 culture wells per data point and are representative of 4 independent experiments. **b** and **d** One-phase exponential decay curves fit for the highest non-cytotoxic concentration of NTZ and AN7973, respectively. **b** No exponential decay curve can be fit for NTZ. **d** Rapid parasite elimination by AN7973. Curves shown are for 3 × EC_90_ (0.63 µM), 12 × EC_90_ (2.5 µM) or the DMSO control. For both **b** and **d**, the data are referenced to the DMSO control at each time point, in order to isolate the effect of compounds from the spontaneous decline in parasite numbers that occurs in this *C. parvum* culture system. Data points are the means and SD for 4 culture wells per time point and are representative of 4 independent experiments

Treatment of cryptosporidiosis in immunocompromised individuals such as malnourished children, AIDS patients, and transplant patients might be expected to require a parasiticidal compound. Consistent with this hypothesis, *C. parvum* time-kill curve assays showed that the approved drug nitazoxanide, which is effective in immunocompetent adults but equivalent to a placebo in AIDS patients, is likely parasitistatic or very slow acting (Fig. 3a)^19^. Time-kill curve data were referenced to the vehicle control for each time point in order to isolate the effect of compounds from the spontaneous decline in *C. parvum* numbers that occurs after 24 to 48 h of in vitro culture. This demonstrated the absence of nitazoxanide-dependent parasite elimination (Fig. 3b). AN7973, on the other hand, appeared to act rapidly and drove progressive elimination of *C. parvum* in vitro in the absence of immune pressure (Fig. 3c, d). In vitro parasite elimination in the presence of AN7973 was exponential with a half-life of ∼9.2 h, and ∼92 h required for 99.9% parasite reduction. The maximum rate of parasite elimination was achieved at ∼3 × EC_90_ (0.63 µM).

### Pharmacokinetic properties of AN7973

In anticipation of progression to mouse and calf models of cryptosporidiosis, we evaluated the pharmacokinetic properties of AN7973 in these species. When administered intravenously to CD-1 mice at a dose of 2 mg per kg as a solution in a polyethylene glycol/propylene glycol/water vehicle, AN7973 exhibited a high C_max_ (6.82 μg per mL), very low clearance (CL = 40 mL•h^−1^•kg^−1^), high exposure (AUC_0-inf_ = 49.7 h•μg^−1^•mL^−1^), and long half-life (t_1/2_ = 6 h). When administered orally to CD-1 mice at a dose of 10 mg per kg as a suspension in 1% CMC/0.1% Tween-80 aqueous vehicle, AN7973 showed very high C_max_ (8.63 μg per mL) and exposure (AUC_0-inf_ = 92.7 h•μg^−1^•mL^−1^), a long half-life (t_1/2_ = 6.6 h) and modest bioavailability (F = 37%) (Supplementary Table 1 and Supplementary Fig. 4a). When dosed orally to calves at a dose of 5 mg per kg in the 1% CMC/0.1% Tween-80 aqueous vehicle, AN7973 exhibited high C_max_ (3.57 μg per mL), very high exposure (AUC_0-inf_ = 190 h•μg^−1^•mL^−1^), and a half-life about five times greater than that seen in mice (t_1/2_ = 31.1 h) (Supplementary Fig. 4c).

In both the mouse and calf PK studies, we also quantified AN7973 in feces, as we expected that at least some of the antiparasitic activity could be related to direct exposure of parasites to the drug in this matrix. In the mouse study, concentrations of AN7973 in feces generally increased over the 16 h period following oral dosing but were highly variable (Supplementary Fig. 4b). In calves dosed orally, considerably lower variability was observed between animals, and concentrations of AN7973 remained high through the entire 168 h observation period (Supplementary Fig. 4c).

### Efficacy in acute and established murine cryptosporidiosis

**Fig. 4:**
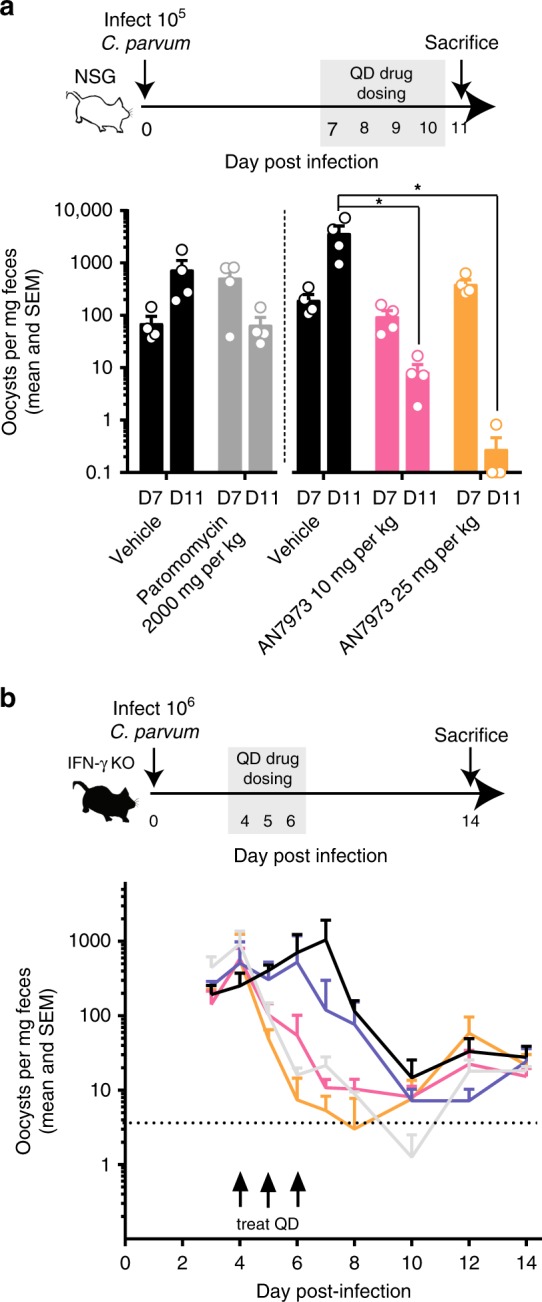
AN7973 efficacy in established and acute murine *C. parvum* infections. **a** Effect of AN7973 on established cryptosporidiosis in NOD scid gamma mice. *C. parvum* (Bunch Grass Farms Iowa isolate) infection was established by oral gavage of ∼10^5^ oocysts and allowed to progress for 7 days prior to once daily oral administration of paromomycin (positive control), or AN7973 at the indicated dosages. Data are the mean and SEM (*n* = 4 mice per experimental group) of parasite fecal shedding per mg of feces measured by qPCR. Note that no oocyst shedding was detected on day 5 for 3 of 4 mice treated with 25 mg per kg AN7973, and data points are shown at the assay’s limit of detection (0.1 oocysts per mg feces). Asterisk (*) indicates *p* ≤ 0.02 by one-way ANOVA test with Dunnett’s Method for multiple comparisons. **b** Effect of AN7973 in an acute, self-resolving IFN-γ knockout mouse model of cryptosporidiosis. *C. parvum* infection was established by oral gavage of ∼10^6^ *C. parvum* (University of Arizona Iowa isolate) oocysts followed by daily oral treatment beginning on day 4 post-infection using the indicated dose of AN7973 or clofazimine (positive control). Lines and dosing regimen are color coded as follows: black line (vehicle); gray line (clofazimine 25 mg per kg); purple line (AN7973 5 mg per kg); pink line (AN7973 10 mg per kg); and orange line (AN7973 25 mg per kg). Data are the mean and SEM (*n* = 4 mice per experimental group) of fecal oocyst shedding as measured by immunostaining and flow cytometry

AN7973 efficacy was first tested in vivo using a NOD scid IL2rg^null^ (NOD scid gamma (NSG)) mouse model, which is a model of established *C. parvum* infection^19^. Treatment was begun 7 days after infecting mice by oral gavage with the *C. parvum* Bunch Grass Farms Iowa isolate. The positive control compound paromomycin reduced fecal parasite shedding by ∼90% after 4 days of treatment. On the other hand, after 4 days of treatment, AN7973 reduced parasite shedding by >99% at a dose of 25 mg per kg and by >90% at a dose of 10 mg per kg administered once daily by oral gavage (Fig. 4a). NSG mice treated with AN7973 gained weight normally, and no adverse effects were observed.

The interferon gamma knockout (IFN-γ KO) mouse is another widely used *Cryptosporidium* animal model^17,39–41^. Unlike lethal infection reported by some investigators, *C. parvum* infection of IFN-γ KO mice produces a self-resolving acute infection in our hands. Various doses of AN7973 were tested using this model and the *C. parvum* University of Arizona Iowa isolate. Clofazimine, which is parasitistatic and lacks efficacy against *Cryptosporidium* in the NSG mouse^19^, was used as a positive control^17^. AN7973 demonstrated dose-dependent efficacy, including efficacy at a dose of 10 mg per kg once daily. Maximal efficacy was similar to that seen with clofazimine (Fig. 4b). As was the case for NSG mice, AN7973 caused no adverse effects in IFN-γ KO mice.

### AN7973 efficacy in neonatal calves

**Fig. 5:**
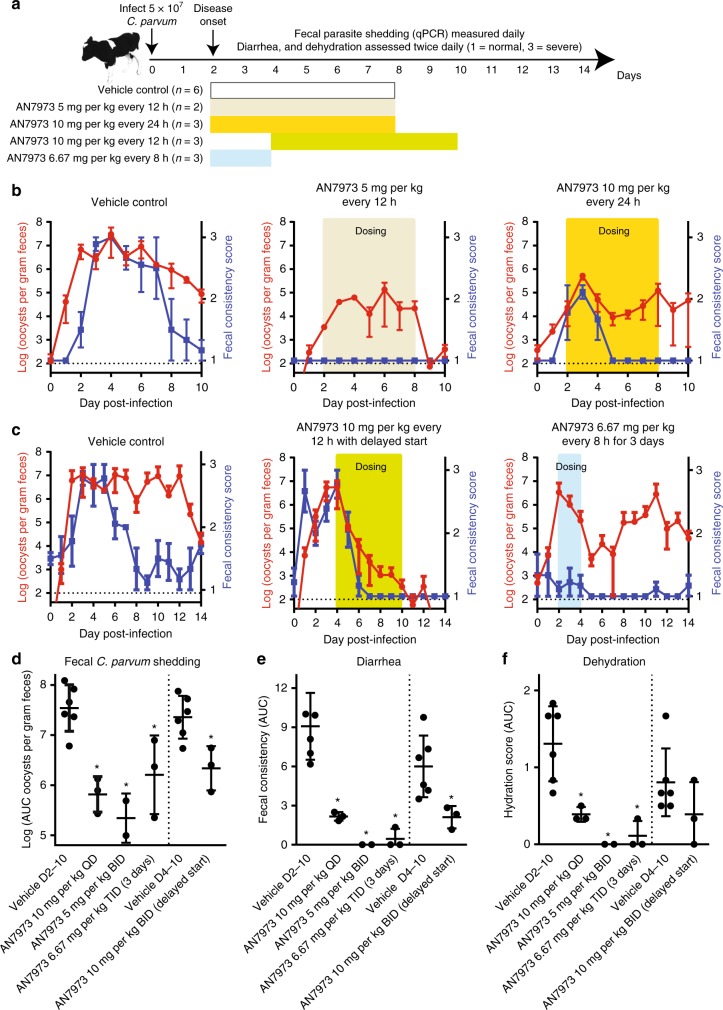
Efficacy of AN7973 in a neonatal calf model of cryptosporidiosis. **a** Summary of calf model and AN7973 microbiologic and clinical efficacy studies conducted. Bull Holstein calves were challenged within 48 h of birth by oral administration of ∼5 × 10^7^ *C. parvum* (Bunch Grass Farms Iowa isolate) oocysts. Fecal oocyst shedding was quantified daily using qPCR, and clinical assessments were conducted two times daily and quantified on a scale of 1 to 3 (1 = normal, 3 = severely abnormal (e.g., severe diarrhea, or severe dehydration)). Treatments were given at the times highlighted in color in **a** to **c**, using the following dosing regimens: no color (vehicle control given on days 2–8); tan (AN7973 5 mg per kg every 12 h on days 2–8); yellow (AN7973 10 mg per kg every 24 h on days 2–8); green (AN7973 10 mg per kg every 12 h on days 4–10); and blue (AN7973 6.67 mg per kg every 8 h on days 2–4). **b** Initial AN7973 calf study. Each graph shows the mean and SEM of fecal oocyst shedding (red) or diarrhea score (blue) vs. time for the indicated treatment regimen (*n* = 3 calves per experimental group, except for *n* = 2 for the 5 mg per kg group). Dotted horizontal lines indicate the approximate limit of detection for the qPCR assay. **c** Follow-up AN7973 calf study, demonstrating clinical and microbiologic efficacy with delayed administration. A 3-day course was effective for diarrhea, but was followed by a relapse of shedding. Each graph shows the mean and SEM of fecal oocyst shedding (red) or diarrhea score (blue) vs. time (*n* = 3 calves per experimental group). Dotted horizontal lines indicate the approximate limit of detection for the qPCR assay. **d** Scatter plot showing the area under the curve (AUC) for Log_10_ transformed fecal oocyst shedding for individual animals. The lines indicate means and SD. Asterisk (*) indicates *p* < 0.02 compared with the corresponding vehicle control by one-way ANOVA with Dunnett’s Correction for multiple comparisons. **e** and **f** Scatter plots showing the AUC for the diarrhea and dehydration scores for individual animals receiving each treatment regimen. The lines indicate means and SD. Asterisk (*) indicates *p* < 0.02 compared with the corresponding vehicle control by one-way ANOVA with Dunnett’s Correction for multiple comparisons

Mice infected with *C. parvum* do not develop diarrhea, but neonatal dairy calves infected with *C. parvum* develop a self-limited illness much like that seen in infants and characterized by severe watery diarrhea, dehydration, and fecal oocyst shedding^42^. We therefore used a neonatal calf model to test the effect of AN7973 treatment on diarrhea and dehydration, in addition to its effect on parasite shedding. For this, an initial small efficacy study was conducted during which the effect of infection on PK was also assessed. These data were then used to guide the design of additional dosing regimens (Fig. 5a). One-day-old to two-day-old bull calves were infected by oral administration of ∼5 × 10^7^ *C. parvum* Bunch Grass Farms Iowa isolate oocysts. Fecal parasite shedding was measured daily using qPCR, and the animals were assessed clinically at least twice daily for diarrhea, hydration status, appetite, and overall health status. Clinical observations were quantified using previously described scales ranging from 1 (normal) to 3 (severely abnormal) for fecal consistency, overall health status, hydration status, and appetite (see Supplementary Table 2)^43^. Finally, the areas under each time course curve (AUC) were calculated to provide an overall measure of the impact of AN7973 on each parameter and facilitate statistical comparison.

All calves tolerated AN7973 well, based on at least twice daily assessment of alertness, interaction with caregivers, and appetite (see Supplementary Table 2 for clinical scoring rubric). The effects of oral 5 mg per kg twice daily AN7973, 10 mg per kg once daily AN7973, or vehicle alone were first compared, each begun 2 days after infection and continued for 7 days. Plasma and fecal exposures during infection were in-line with predictions based on results in uninfected calves, although fecal levels varied widely on the first day of dosing (Supplementary Fig. 5). Twice daily 5 mg per kg AN7973 curtailed parasite shedding and completely eliminated diarrhea (Fig. 5b, d and e). Once daily 10 mg per kg AN7973 also reduced peak parasite shedding and diarrhea. However, despite equal or better mean plasma exposures in animals treated with once daily 10 mg per kg vs. twice daily 5 mg per kg AN7973, fecal parasite shedding resumed and persisted in the once daily treated animals (Fig. 5b). Based on these data, it was unlikely that plasma exposures alone were driving efficacy, suggesting that gastrointestinal exposure to AN7973 was important.

Major questions addressed in a second calf study included whether the dosing duration could be shortened, and whether or not fully sick animals would respond to treatment. These questions were addressed, respectively, by testing a 3-day regimen of 6.67 mg per kg three times daily, and testing the efficacy of 10 mg per kg twice daily for 7 days when treatment was delayed until the peak of diarrhea. Even when administered beginning at the peak of symptoms on day 4 after infection, twice daily 10 mg per kg AN7973 cured diarrhea 3 days faster than in controls, and parasite shedding was abolished by day 14 (Fig. 5c). Total parasite shedding was reduced by greater than 1 log (Fig. 5d). Treatment with 6.67 mg per kg three times per day for 3 days eliminated diarrhea, and reduced the rate of parasite shedding by ∼3 log during treatment. However, a parasitological relapse followed (Fig. 5c), suggesting that >3 days and ≤7 days of treatment will be required. Finally, several of the AN7973 treatment regimens significantly reduced the overall severity of dehydration (Fig. 5f), a surprising result given that all of the animals received aggressive treatment to minimize dehydration.

### Safety profiling

AN7973 possessed favorable stability, PK (e.g., mouse t_1/2_ = 6 h), and safety characteristics (Supplementary Table 1 and Supplementary Table 3). It was negative in the Ames test for mutagenic potential, was negative in an in vitro micronucleus genotoxicity assay, had no significant findings in Ricerca Bioscience’s Lead Profiling and Adverse Reaction Enzyme off-target-panel (total # of assays: 109), and was negative for inhibition of the hERG K^+^ ion channel, which is associated with cardiac tachyarrhythmias.

Administration of AN7973 by daily oral gavage to male rats at 80 mg per kg per day for seven days was well tolerated, and there were no clinical signs (e.g., changes in activity or grooming (see methods)), weight loss, changes in food consumption, or pathology observed. Slight changes to red blood cell parameters, serum alkaline phosphatase, and serum triglycerides occurred, which were not considered clinically significant. No compound accumulation was observed in a toxicokinetic analysis done in parallel (Supplementary Table 3). Based on these data, the no observed adverse effect level (NOAEL) of AN7973 was concluded to be greater than 80 mg per kg per day in rats. Additional toxicology studies, including higher doses, will be needed to ascertain the complete pre-clinical picture for AN7973.

## Discussion

These studies collectively identify AN7973 as a promising candidate for a new cryptosporidiosis treatment. AN7973 is active in vitro against both *C. hominis* and *C. parvum*, and unlike compounds such as nitazoxanide that lack efficacy in severely immunocompromised mice and people, AN7973 rapidly eliminates *C. parvum* in vitro and is efficacious in immunocompromised mice. Thus, it may be useful for treatment of all patient populations affected by cryptosporidiosis, including AIDS patients, transplant recipients, and malnourished children. In addition to efficacy in mouse models of both acute and established infection, AN7973 reduced *C. parvum* fecal shedding, diarrhea, and dehydration in a neonatal calf model in which disease closely mimics that seen in infants. AN7973 also meets many of the stability and safety requirements for a novel anticryptosporidial^44^, although additional toxicology and PK studies of AN7973 are needed. Note that AN7973 was 3-fold to 4-fold less potent in vitro against the *C. hominis* TU502 isolate, which highlights the need to test in vivo efficacy of AN7973 against *C. hominis*.

The anticryptosporidial mechanism of action of AN7973 remains to be experimentally determined. The EdU incorporation assay (Fig. 2c) results demonstrate only that AN7973 arrests intracellular *Cryptosporidium* development prior to DNA synthesis, and do not imply that the compound directly affects DNA synthesis. Many compounds that do not directly target DNA synthesis (e.g., several tRNA-synthetase inhibitors presumed to inhibit protein synthesis) look similar to AN7973 in this assay^38^. Recent studies of AN7973 demonstrated that its trypanicidal activity results from inhibition of trypanosome mRNA processing^45^. Furthermore, related HAT and AAT lead compounds, also 6-amides like AN7973, target Cleavage and Polyadenylation Specific Factor 3 (CPSF3), an endonuclease that functions in mRNA processing^46^. It is known that other oxaboroles inhibit CPSF3 in *Plasmodium falciparum*^28^, and *Cryptosporidium* CPSF3 is greater than 40% identical to that of *P. falciparum* at the amino acid level. It therefore seems possible that AN7973 inhibits *Cryptosporidium* CPSF3 as well.

Given the modest efficacy of nitazoxanide, improved drugs to treat cryptosporidiosis in the most vulnerable populations (i.e., malnourished children and immunocompromised people) are eagerly awaited by clinicians^47^. Multiple lead compound series that affect a variety of targets may be needed, given the high attrition rate that is typical during drug development. This may also help to address the inevitable issue of drug resistance, either simply by providing alternative drugs or by facilitating development of drug combinations to reduce the likelihood that resistance will evolve. Towards this goal, phenotypic screening assays have been developed and a growing number of lead compounds have been reported^17–19,32,48–52^. The phosphatidyl-inositol 4 kinase (PI4K) inhibitor KDU731, which was repurposed from a malaria drug development effort, is currently the most advanced compound in the *Cryptosporidium* pipeline^16^.

AN7973 reduced the fecal *C. parvum* shedding rate by nearly five logs in the first three days of treatment in the neonatal calf model, which we believe is the most rapid effect yet reported in this model^16,18,43^. Furthermore, AN7973 treatment reduced the total burden of fecal *C. parvum* shedding (i.e., the AUC) by over 90%. Based on its efficacy, stability, and preliminary in vitro and rat safety characteristics, AN7973 is amongst the most advanced compounds in the current pipeline, and is poised for pre-clinical toxicology studies in hopes of advancement to human clinical trials.

## Methods

### PCR primers

Where noted, the following primers were used for quantitative PCR amplification of *C. parvum* 18s rRNA: Cp18s forward-TAGAGATTGGAGGTTGTTCCT, and Cp18s reverse-CTCCACCAACTAAGAACGGCC^53^.

### Compound characterization

The chemical identity of AN7973 was established through ^1^H NMR, ^13^C NMR, and high-resolution mass spectral (HRMS) analysis. Compound purity was assessed by high performance liquid chromatography (HPLC) (Instrument and column: HPLC-01 Venusil MP C18 5 µm 4.6 × 50 mm).

### In vitro activity of compounds against *Cryptosporidium*

A high-content microscopy assay was used to measure activity of compounds against *C. parvum*^32,54^. Screening was performed at the University of California San Francisco using Madin-Darby canine kidney (MDCK) type 2 (ATCC CRL-2936) cells grown to confluence in clear-bottomed 384-well plates in 50 µL per well of Dulbecco’s minimum essential medium (DMEM; Life Technologies) with 5% heat inactivated fetal bovine serum (FBS). The concentration of FBS was reduced to 1% prior to the addition of parasites. *C. parvum* Iowa isolate oocysts used for screening were purchased from the Sterling Laboratory at the University of Arizona and stored at 4 °C until use. Oocysts were induced to excyst by treatment for 10 min at 37 °C with 10 mM HCl and then 10 min at 15 °C in 2 mM sodium taurocholate, washed in the above medium with 1% FBS, and then added to cell monolayers in the presence of compounds. The number of oocysts added to each well was dependent on the viability of the parasite stock, and varied between experiments. Compounds were screened at a concentration of 1 µM. After incubation at 37 °C under 5% CO_2_ for 48 h, the cells were fixed for at least 24 h by adding an equal volume of 8% formaldehyde in PBS. After fixation, the cells were blocked with 0.1% Titron X-100, 0.25% bovine serum albumin (BSA) in saline, which was also used for all subsequent steps. Cells were incubated with 0.5 µg per mL of biotinylated *Vicia villosa* lectin (VVL, Vector Laboratories, catalog# FL-1231) for 1 h at room temperature, washed three times, and stained in the dark for 1 h at room temperature in 0.5 µg per mL Cy3-streptavidin (Jackson ImmunoResearch, catalog# 016-160-084) containing 1 nM DAPI [2-(4-amidinophenyl)-1*H*-indole-6-carboxamide]. After incubation, the plates were washed three times and 50 µL per well of the above blocking buffer was added. Plates were stored at 4 °C in the dark until processed. Images were acquired using a GE InCell 2000 automated microscope, and GE InCell Developer (version 1.9) image analysis software was used to quantify the parasites and host cells. Confirmation by 8-point dose response curve was performed in triplicate, with half-log serial dilutions starting at 10 µM.

Follow-up assays were performed at the University of Vermont (UVM) and Calibr at Scripps Research by infection of the colonic adenocarcinoma cell line HCT-8 (ATCC CCL-244) and staining with the *Vicia villosa* lectin and DAPI^17,32^. The FBS concentrations and parasites used for these assays were as indicated; parasite isolates and sources included *C. parvum* Iowa isolate oocysts purchased from Bunch Grass Farm (Deary, ID), *C. parvum* Iowa isolate oocysts purchased from the Sterling Laboratory (University of Arizona, Tucson, AZ), wild type *C. parvum* oocysts provided by Dr. Jenni Zambriski (Virginia Tech, Blacksburg, VA), and the *C. hominis* TU502 isolate purchased from Dr. Saul Tzipori (Tufts University Cummings School of Veterinary Medicine, North Grafton, MA).

### Cell-based mode of action and time-kill curve assays

The invasion assay, DNA replication assay, asexual to sexual stage conversion, and time-kill curve assays were performed using fluorescence microscopy to enumerate parasites and host cell nuclei. Staining protocols are individually described for each assay. Unless noted otherwise, the details of image acquisition were as follows. An inverted Nikon Eclipse Ti2000 microscope with an automated stage and Perfect Focus was used with NIS-Elements Advanced Research software (Nikon, USA, version 4.30.01) to image the wells. The microscope was programmed to focus on the center of each well and then take a 3 × 3 composite image per well using a 20 × (0.45 NA) objective. Images were exported as .tiff files and parasites and host nuclei counted using a custom macro on ImageJ (National Institutes of Health, version 1.52a). Data were analyzed using Microsoft Excel and GraphPad Prism (version 6.01).

Host cell invasion was assayed by allowing *C. parvum* to invade host cell monolayers in the presence of compound, and enumerating parasites and host cells after just 3 h (i.e., before completion of a parasite division cycle)^38^. HCT-8 cells were grown in RPMI media supplemented with 120 U per mL penicillin and 120 μg per mL streptomycin and 10% heat inactivated fetal bovine serum (complete media) and *C. parvum* Iowa isolate oocysts from Bunch Grass Farms (Deary, ID) were used for infection. When cells reached a confluence of >99%, media was removed and replaced with 25 µL per well of fresh media containing twice the indicated concentrations of compounds and incubated for 1 h at 37 °C in a humidified CO_2_ incubator. In the meantime, oocysts were induced for excystation by first treating with 10 mM hydrochloric acid (diluted with distilled water) for 10 min at 37 °C followed by 2 mM sodium taurocholate (in phosphate buffered saline (PBS) with calcium and magnesium) for 10 min at 16 °C. Before and after each treatment, oocysts were spun down at 14,000 × *g* for 4.5 min and supernatant was removed. Oocysts were then diluted in complete media and added to cells at 5 × 10^4^ oocysts in 25 µL per well such that the final concentration of compounds was the same as the indicated concentration. Oocysts were allowed to excyst and sporozoites allowed to invade in presence of indicated compound concentrations for 3 h after which cells were washed three times with PBS containing 111 mM D-galactose, fixed with 4% formaldehyde for 15 min at room temperature, followed by permeabilization with 0.25% triton X-100 for 10 min at 37 °C. Cells were then washed three times with PBS containing 0.1% tween 20 and blocked with 4% bovine serum albumin (BSA) in PBS overnight at 4 °C. Invaded parasites were stained with 1.33 µg per mL of fluorescein-labeled *Vicia villosa* lectin (Vector Laboratories, catalog# FL-1231) for 1 h at 37 °C followed by nuclei staining with 0.09 mM Hoechst 33258 (AnaSpec, catalog# AS-83219) for 15 min at 37 °C and then washed five time with PBS containing 0.1% tween 20. Each of the negative controls were treated the same, except for one of the following additional pre-steps. Heat killed oocysts were first heat killed at 70 °C for 30 min before being induced for excystation. For fixed host cells, HCT-8 cells seeded in 384 well plates at >99% confluence were fixed with 4% formaldehyde for 15 min at room temperature, followed by 3 washes with warm complete media before the experiment.

Measurement of DNA replication was used as a surrogate marker of *Cryptosporidium* development within HCT-8 cells^38^. For this, glass bottom 96-well plates were coated with 50 µg per mL fibronectin (BD Pharmingen, catalog# 354008) according to the manufacturer’s protocol, and then HCT-8 cells were grown to >90% confluence in the wells. All mode of action and time-kill curve assays were performed in 10% FBS. *C. parvum* Iowa isolate oocysts (Bunch Grass Farms, Deary, ID) were triggered for excystation and added at a density of 5.5 × 10^4^ per well. After a 3-h delay, AN7973 was added at ∼2 × EC_90_ (0.42 µM) followed by incubation for 6 h and then addition of 10 mM 5-ethynyl-2′-deoxyuridine (EdU). After incubation with EdU for 2 more hours, the cells were washed and fixed with 4% formaldehyde in saline. Cells were then permeabilized and stained for incorporation of EdU using the Click-iT assay kit (Thermo Fisher Scientific, catalog# 10340). Images were acquired by focusing on the parasite focal plane, which is on top of the host cell monolayer, using a ×40 objective (0.7 NA) and EdU and lectin numbers were quantified using ImageJ software.

The effect of AN7973 on asexual vs. sexual stage *Cryptosporidium* was assessed by comparing its potency for reducing the percent of host cells infected during the first 48 h of development within HCT-8 cells, which reflects asexual parasite development, to its effect on expression of the meiotic recombination protein DMC1, which is a previously identified sexual stage-specific marker^38^. For this, AN7973 was added after 48 h of culture (the approximate timing of sexual differentiation) and its effect on appearance of DMC1 positive parasites was measured using high-content microscopy, after staining with FITC-conjugated *Vicia villosa* lectin and DAPI as above, and with an anti-*C. parvum* DMC1 mouse monoclonal antibody (clone 1H10G7 (IgG2b, kappa) used as undiluted culture supernatant) with a secondary Alexa Fluor 568 goat anti-mouse IgG antibody (Invitrogen, catalog# A-11004) at 1:500 dilution (4 µg per mL). Amphotericin B (Sigma, catalog# A2942) was added to the culture media at 0.1 to 0.5 µg per mL for the DMC1 dose response assays.

Time-kill curve assays were used to assess if AN7973 is parasiticidal or parasitistatic for *C. parvum*^19^. *C. parvum* Iowa isolate oocysts (Bunch Grass Farms, Deary, ID) were treated to induce excystation and added to >90% confluent HCT-8 cells in 384-well plates. AN7973 was added at the EC_50_ (0.13 µM) or multiples of the EC_90_ (0.21 µM) ∼24 h after infection. The monolayers were then washed, fixed, and stained with *Vicia villosa* lectin and DAPI at the indicated time points, and imaged as for the *C. parvum* growth assay. Separate 384-well plates were used for each time point. Parasite numbers were normalized to host cell nucleus numbers and expressed as the percentage of host cells infected. Drug-induced parasite decay was isolated from the reduction in parasites over time that occurs spontaneously in the HCT-8 culture system by expressing the number of parasites as the percentage of the number of parasites present at each time point under control (DMSO-treated) conditions.

### Pharmacokinetic studies

Single-dose oral and intravenous murine PK studies were performed by WuXi Biologics (Wuxi, China) in female CD-1 mice in compliance with animal care guidelines. AN7973 was formulated at 1 mg per mL in 50/20/30 PEG300/propylene glycol (PG)/water for IV administration, and at 2 mg per mL in 1% carboxymethylcellulose in water with 0.1% Tween 80 for oral administration. Single-dose oral calf PK studies were performed at the University of Vermont in compliance with animal care guidelines and with approval from the University of Vermont Institutional Animal Care and Use Committee. Holstein bull calves were acquired within 24 h of birth and group-housed. AN7973 was formulated as a suspension at 25 mg per mL in 1% carboxymethylcellulose in water with 0.1% Tween 80, which was then diluted with water to achieve a concentration needed for a 10 mL dose. Doses were squirted into the calves’ mouths during interruptions in bottle feeding. Fecal samples were collected at the indicated times by manual anal stimulation. For both mouse and calf studies, plasma samples were analyzed for AN7973 by liquid chromatography-tandem mass spectrometry (LC-MS/MS); sample concentrations were calculated using a standard curve of compound added to control plasma. Fecal AN7973 was measured by extracting AN7973 from homogenized feces with acetonitrile. For this, feces were added to 4 volumes of acetonitrile (4 mL per g), mixed by vortexing for 5 min at room temperature, and then centrifuged. One hundred microliter samples were then dried under nitrogen, reconstituted in 250 µL 50/50 acetronitrile/water, and analyzed by LC-MS/MS. The fecal extraction method was validated with a standard curve of compound added to control feces.

AN7973 mean plasma and feces concentrations and nominal time were used to construct semi-logarithmic plasma concentration versus time curves (Supplementary Fig. 4). Non-compartmental analysis of the mean concentration-time profiles was performed using Phoenix version 6.4 (Pharsight Corporation, Mountain View, CA, USA). Concentrations below the limit of quantitation were assigned values of zero for generation of mean concentrations. Predictions of multidose exposures were generated by non-parametric superposition of the single-dose PK data using Phoenix version 6.4 (Supplementary Fig. 5).

### Murine models of cryptosporidiosis

NOD scid gamma mice (NOD.Cg-Prkdcscid Il2rgtm1Wjl/SzJ)^55^ (NSG) were used to model chronic *Cryptosporidium* infection^19^. All NSG mouse studies were performed in compliance with animal care guidelines and with approval by the University of Vermont Institutional Animal Care and Use Committee. NSG mice with normal flora were purchased from The Jackson Laboratory (Bar Harbor, ME) and were housed for at least a week for acclimatization. At the age of four to five weeks, mice were infected by oral gavage with ∼10^5^ *C. parvum* Iowa isolate (Bunch Grass Farms) oocysts. On days 7 to 11 following infection, mice were treated once daily by oral gavage with AN7973 or paromomycin (positive control) at the indicated doses with each compound dissolved in DMSO and diluted with 1% hydroxypropyl methylcellulose (HPMC) to a final concentration of 5% DMSO and final volume of 100 µL. Fecal parasite shedding was measured using a quantitative PCR (qPCR) assay, and the primers named above as Cp18s forward and Cp18s reverse^53^.

Interferon gamma (IFN-γ) knockout mice were used to model acute *Cryptosporidium* infection^19^. All IFN-γ knockout mouse studies were performed in compliance with animal care guidelines and were approved by the Explora BioLabs (San Diego, CA) Institutional Animal Care and Use Committee. Four-week-old female C57BL/6 IFN-γ^−/−^ mice with normal flora were purchased from The Jackson laboratory and acclimated for 3 days prior to infection by oral gavage with ∼10^6^ *C. parvum* Iowa isolate oocysts (Sterling Laboratory, University of Arizona) suspended in sterile distilled water. At the indicated times following infection, mice were treated with compound vehicle alone, clofazimine (positive control), or once daily AN7973 at the indicated doses. Fecal parasite shedding was measured by isolating oocysts using sucrose gradient centrifugation. Sheather’s sucrose solution was freshly prepared by dissolving 156.25 g sucrose and 2.5 mL phenol in 100 mL water. Two sucrose solutions were prepared: (A) specific gravity of 1.103 (33% Sheather’s and 0.1% Tween 80 in PBS) and (B) specific gravity of 1.064 (20% Sheather’s and 0.1% Tween 80 in PBS). In a 2 mL microfuge tube, a gradient was prepared by laying 0.75 mL of solution A, then carefully laying 0.75 mL of solution B, slowly as to prevent mixing of the two layers. Fecal pellets were removed from storage, and homogenized by vortexing and pipetting. 0.2 mL fecal homogenate was overlaid on top of gradient in each microfuge tube. Oocysts were floated from feces by centrifugation at 1000 × *g* for 20 min and collected with a pipet tip from the 1.064/1.103 specific gravity interface between solutions A and B. Oocysts were rinsed once in cold PBS, pelleted by centrifugation at 15,000 × *g* for 10 min, and resuspended in PBS. Isolated oocysts were then stained with a fluorescein isothiocyanate-conjugated mouse anti-*Cryptosporidium* antibody (Bio-Rad catalog# 2402-3007 F; 0.25 µg per sample) and quantified using a Guava EasyCyte flow cytometer and CytoSoft data acquisition software (version 5.3; Guava Technologies, Inc.). Oocysts per milliliter of sample were normalized to counts per milligram of feces.

### Neonatal calf model of cryptosporidiosis

All calf efficacy studies were performed as previously described^43^. These studies were approved by the University of Vermont Institutional animal Care and Use Committee, and conducted in compliance with the USDA-APHIS “Blue Book”, available at www.aphis.usda.gov/animal-welfare. Holstein bull calves were acquired at birth from Green Mountain Dairy (Sheldon, VT), given synthetic colostrum with 200 g of IgG (Land O’Lakes, Ardent Hills, MO) and bovine coronavirus and *Escherichia coli* antibodies (First Defense Bolus, Immuncell Corporation, Portland, ME) within 2 h of birth, and transported to UVM. Calves were group-housed initially, and infected at 24–48 h of age during an interruption in bottle feeding by oral administration of ∼5 × 10^7^ *C. parvum* Iowa isolate oocysts (Bunch Grass Farms, Deary, ID) suspended in 10 mL of water. They were then moved to individual raised pens, and observed twice daily at feeding times for clinical signs, which were quantified according to a previously described standardized scoring rubric^43^. Data on fecal consistency, dehydration and overall health were collected, in each case with a score of 1 being normal and 3 being severely abnormal (scoring rubric in Supplementary Table 2). Clinical microbiologic studies for adventitious infectious agents including *Salmonella* culture, aerobic bacterial culture with *E. coli* genotyping, rotavirus, and coronavirus testing were performed on all calves at the onset of diarrhea by the Cornell Animal Health Diagnostic Center (Ithaca, NY). Animals with severe diarrhea or other symptoms were supported aggressively, including with administration of oral electrolytes, intravenous fluids and flunixin meglumine (Banamine, Merck) as needed. AN7973 was suspended as for the PK study in 1% carboxymethylcellulose in water with 0.1% Tween 80 at a final volume of 10 mL per dose, and was administered orally by squirting doses into the calves’ mouths during interruptions in bottle feeding. Daily fecal samples were obtained from collections bins located under each pen. Fecal samples used for parasite quantification were dried at 90 °C until stable in weight, and *C. parvum* abundance per gram of fecal dry matter was measured using qPCR and the primers Cp18s forward and Cp18s reverse^53^.

### In vitro safety profiling and rat toxicology

In vitro safety profiling studies were conducted by Ricerca Biosciences (Taipei, Taiwan) and WuXi AppTec (Suzhou, China). Ricerca performed a Lead Profiling and Adverse Reaction Enzyme panel of 109 off-target enzymes and receptors. This included the hERG K+ ion channel, which is associated with cardiac tachyarrhythmias, and used a radioligand binding competition format with 10 µM test compound for 1 h at 25 °C. For the enzyme and receptor panel, competitive inhibition at 10 µM of test compound was the most common assay format. The AMES bacterial reverse mutation assay detects point mutations in *Escherichia coli* and *Salmonella typhimurium* as an indicator of mutagenic activity. Bacterial strains were obtained from Molecular Toxicology (Boone, NC). WuXi tested AN7973 against five strains with and without S9 (Aroclor 1254 induced rat liver S9) in triplicate. AN7973 was tested for mutagenic activity at doses ranging up to 5000 µg per plate. An in vitro micronucleus genotoxicity assay was performed to assess the clastogenic/aneugenic potential of AN7973. WuXi tested AN7973 against human lymphocytes with and without S9 for 3 and 28 h using a range of concentrations from 0.25 to 260 µg per mL, in duplicate, and observed by microscopy for induction of micronucleated human lymphocytes.

A repeated oral dose toxicity and separate toxicokinetics (TK) study were performed in male rats by WuXi AppTec Company (Suzhou, China). These studies were reviewed and approved by the WuXI AppTec Institutional Animal Care and Use Committee, and a staff veterinarian monitored them for animal welfare issues. Animals were individually housed in solid bottom plastic cages with bedding in compliance with the US Animal Welfare Act. All rat studies were performed using ∼8-week-old Sprague-Dawley male rats (*Rattus novegicus*)/Crl: CD[SD] VAF/Plus weighing between 243.22 and 276.57 g at the start of compound dosing. A 7-day repeated oral dose toxicity study was performed using 5 vehicle (0.5% (w/v) methyl cellulose and 0.1% (w/v) sodium dodecyl sulfate in purified water) control and 5 treatment group rats dosed AN7973 80 mg per kg once daily oral gavage for 7 consecutive days. Cage side clinical observations of condition and behavior (i.e., gait, stool, activity level, atonia, coat soiling and grooming, skin turgor, posture, priapism, piloerection) were performed twice daily during the dosing phase, and weights and food consumption were measured daily. At termination, all study animals were evaluated for erythrocyte count, absolute reticulocyte count, alkaline phosphatase, and triglycerides by fasting the animals overnight and obtaining blood from the abdominal aorta at necropsy as a terminal procedure under deep anesthesia (pentobarbital). A complete necropsy and histopathologic studies (hematoxylin and eosin staining) of large intestine, small intestine, stomach, and any gross lesions were performed on all animals on day 7 of dosing. A TK study was performed in parallel to determine exposure, using 3 vehicle control animals and 6 experimental animals dosed 80 mg per kg AN7973 by oral gavage once daily for 7 consecutive days. Serum was collected from vehicle-control animals at 1 h and 4 h after dosing on days 1 and 7. The AN7973 recipient group was divided into two, with serum collected according to the following schedule: first 3 of 6 animals (pre-dose, 1 h, 4 h, 24 h, and 72 h post dosing on days 1 and 7 (72 h time point day 7 only)); and second 3 of 6 animals (0.5 h, 2 h, 8 h, 48 h post dosing on days 1 and 7 (48 h time point day 7 only)). Following completion of blood collection, TK animals were euthanized by CO_2_ inhalation and exsanguination without necropsy. AN7973 was quantified using a qualified liquid chromatographic triple quadruple mass spectrometric (LC-MS/MS) method that had a lower limit of quantification of ≤5 ng per mL.

### Statistical information, and figure preparation

GraphPad Prism software (version 6.01) was used to prepare all graphs, calculate compound EC50 values, and perform statistical analyses. The limit of detection (LOD) of the qPCR fecal oocyst detection assay is ∼100 oocysts per gram of dried feces, and samples for which no signal was detected were set to the LOD for purposes of graphing and statistical analysis. Where indicated, statistical significance was assessed using a one-way ANOVA test with Dunnett’s Method for multiple comparisons. Graphs were exported as .eps files, and final figures were prepared using Adobe Illustrator CS5.

### Reporting summary

Further information on research design is available in the Nature Research Reporting Summary linked to this article.

## Supplementary information

Supplementary Information

Reporting Summary

Source Data

## Data Availability

All data generated and/or analyzed during the study of AN7973 are available from the corresponding author, C.D.H., upon request. The source data underlying Figs. 2, 3, 4, 5, and Supplementary Fig. 3 are provided as a Source Data File.
