# Supplementary material for: Identification of a potent benzoxaborole drug candidate for treating cryptosporidiosis
Source: Nat Commun. 2019 Jun 27;10:2816. doi: 10.1038/s41467-019-10687-y (PMC6597546; doi:10.1038/s41467-019-10687-y)
Supplement: Supplementary file 1 — Supplementary Information [file 41467_2019_10687_MOESM1_ESM.pdf]

Supplementary Table 1: AN7973 physicochemical, metabolic, PK, and toxicity characteristics. Abbreviations: molecular weight (MW), total polar surface area (tPSA), multidrug resistance (MDCK-MDR), area under the curve (AUC), per oral (PO), cytochrome p450 (CYP450), human Ether-a-go-go Related Gene (hERG).

**Physicochemical properties**	
MW	382
ClogD@ pH 7.4	3.7
tPSA	76
***In vitro* ADME properities**	
Liver microsome Cl_{int} ($\mu L \cdot min^{-1} \cdot mg^{-1}$)	<1 (mouse); <1 (human)
MDCK-MDR1 P_{app} ($cm \cdot sec^{-1}$)	56.5
MDCK-MDR1 P_{app} + Pgp inhibitor ($cm \cdot sec^{-1}$)	60.6
Solubility PBS pH 7.4 (mM)	126
Plasma Protein Binding (PPB) (%)	98.9 (mouse); 96 (bovine)
Primary metabolite	Oxidative deboronated product (AN10531) < 1% parent
***In vivo* PK (mouse PO 10 mg·kg^{-1}; bovine PO 5 mg·kg^{-1})**	
$AUC_{0\text{-}last}$ ($hr \cdot \mu g \cdot mL^{-1}$)	92.7 (mouse); 190 (bovine)
% F PO	37 (mouse)
$t_{½}$ (hrs)	6.6 (mouse); 31 (bovine)
**Safety Pharmacology & Genotoxicity**	
CYP450 inhibition (in 6 isoforms – 2B6, 1A2, 2C9,2C19, 2D6, 3A4)	Negative @ 10 µM<br>▪ CYP450 1A2 = 1%<br>▪ CYP450 2C19 = 27%<br>▪ CYP450 2D6 = 0%<br>▪ CYP450 3A4 = 1%
In vitro Receptor Panel (109 targets, 10 µM)	Negative
hERG	Negative
Ames	Negative
In vitro micronucleus	Negative
**Cytotoxicity**	
IC_{50} (µM) THP-1 (3d), Jurkat (3d), and murine L929 (3d)	>25 µM

Supplementary Table 2: Calf study clinical scoring rubric. Definitions used to score clinical signs and symptoms, including fecal consistency, overall health, hydration, and appetite.

**Score**	**Fecal Consistency**	**Health Status**	**Hydration Status**	**Appetite**
**1**	Normal feces: feces retain form	Normal: alert, hungry, interacts with caregivers	Normal: skin tents <1 second; moist mucus membranes	Normal: interacts with caregivers, and eats enthusiastically
**2**	Mild-to-moderate diarrhea: unformed feces; flows down a surface, while leaving some residue	Mildly depressed: some loss of interest in feeding	Mildly dehydrated: skin tents 1-4 seconds; normal mucus membranes	Mild anorexia: some loss of interest in eating, but eats 25-75% of meal
**3**	Severe diarrhea: very watery; flows down a surface, while leaving no residue	Severely depressed: lethargic, must be coaxed to get up, anorexia, requires supportive treatment	Severely dehydrated: skin won't flatten when tented, eyes sunken, dry mucus membranes	Anorexic: loss of interest in feeding; eats 0-25% of meal

Table adapted from Stebbins, EE, et al. Clinical and microbiologic efficacy of the piperazine-based drug lead MMV665917 in the dairy calf cryptosporidiosis model. *PLoS Negl Trop Dis* **12**, e0006183 (2018).

Supplementary Table 3: Summary of 7-day AN7973 rat toxicology study. AN7973 or vehicle were administered by oral gavage at 80 $mg \cdot kg^{-1}$ once daily on 7 consecutive days. Animals were observed daily for behavior, weight change, food consumption. Hematology and serum chemistries were assessed on day 7, followed by full necropsy and histopathologic exam. A toxicokinetic study was performed in parallel to measure AN7973 exposure. Quantified data are given as mean, or mean and standard deviation.

	**Vehicle (n=5)**	**AN7973 (80 $mg \cdot kg^{-1} \cdot d^{-1}$ (n=5))**
**Mortality (unscheduled deaths)**	0	0
**Clinical observations**		
General	No clinical signs noted	No clinical signs noted
Average weight on day 1 (g)	259.47 ± 7.67	260.24 ±7.49
Average weight on day 7 (g)	301.68 ± 9.35	305.36 ± 8.8
Total food consumption (days 1-7)(g)	147.7 ± 13.3	156.1 ± 10.1
**Hematology**		
Red blood cell count ($\times 10^6$ per µl) on day	7.15 ± 0.36	305.6 ± 44.6
Reticulocyte count ($\times 10^9$ per L) on day 8	6.51 ± 0.21	563.2 ± 66.6
**Serum Chemistry**		
Alkaline phosphatase ($U \cdot L^{-1}$) on day 8	257 ± 51	188 ± 17
Triglycerides ($mmol \cdot L^{-1}$) on day 8	0.45 ± 0.08	0.39 ± 0.16
**Toxicokinetic analysis**		**(n=6)**
C_{max} ($ng \cdot mL^{-1}$)	Day 1	11600
	Day 7	10100
T_{max} (h)	Day 1	8.0
	Day 7	8.0
AUC ($h \cdot ng \cdot mL^{-1}$)	Day 1	170000
	Day 7	155000
$T_{1/2}$ (h)	Day 1	NR
	Day 7	22.7
Accumulation indices		
C_{max} ratio	Day 1/Day 7	0.87
$AUC_{0\text{-}24h}$ ratio		0.91

NR: Not reportable due to the lack of quantifiable samples in the terminal phase

a

^{1}H NMR (400 MHz, DMSO-d$_6$): δ 10.56 (1 H, s), 9.11 (1 H, s), 8.69 (1 H, s), 8.10 (2 H, m), 7.98 (1 H, d, J = 5.2 Hz), 7.84 (1 H, s), 7.74 (2 H, m), 7.40 (1 H, d, J = 8.0 Hz), 6.62 (1 H, s), 1.46 (6 H, s).

b

^{13}C NMR (100 MHz, DMSO-d6): δ 163.1, 156.6, 140.1, 139.9, 136.6, 133.1, 130.2,129.1, 128.9, 127.3, 121.8, 120.5, 119.7, 117.6, 115.5, 107.6, 81.2, 28.2.

c

HRMS for $C_{19}H_{18}BClN_3O_3$ $(M+H)^+$: Calc m/z 382.1124; Found m/z 382.1126.

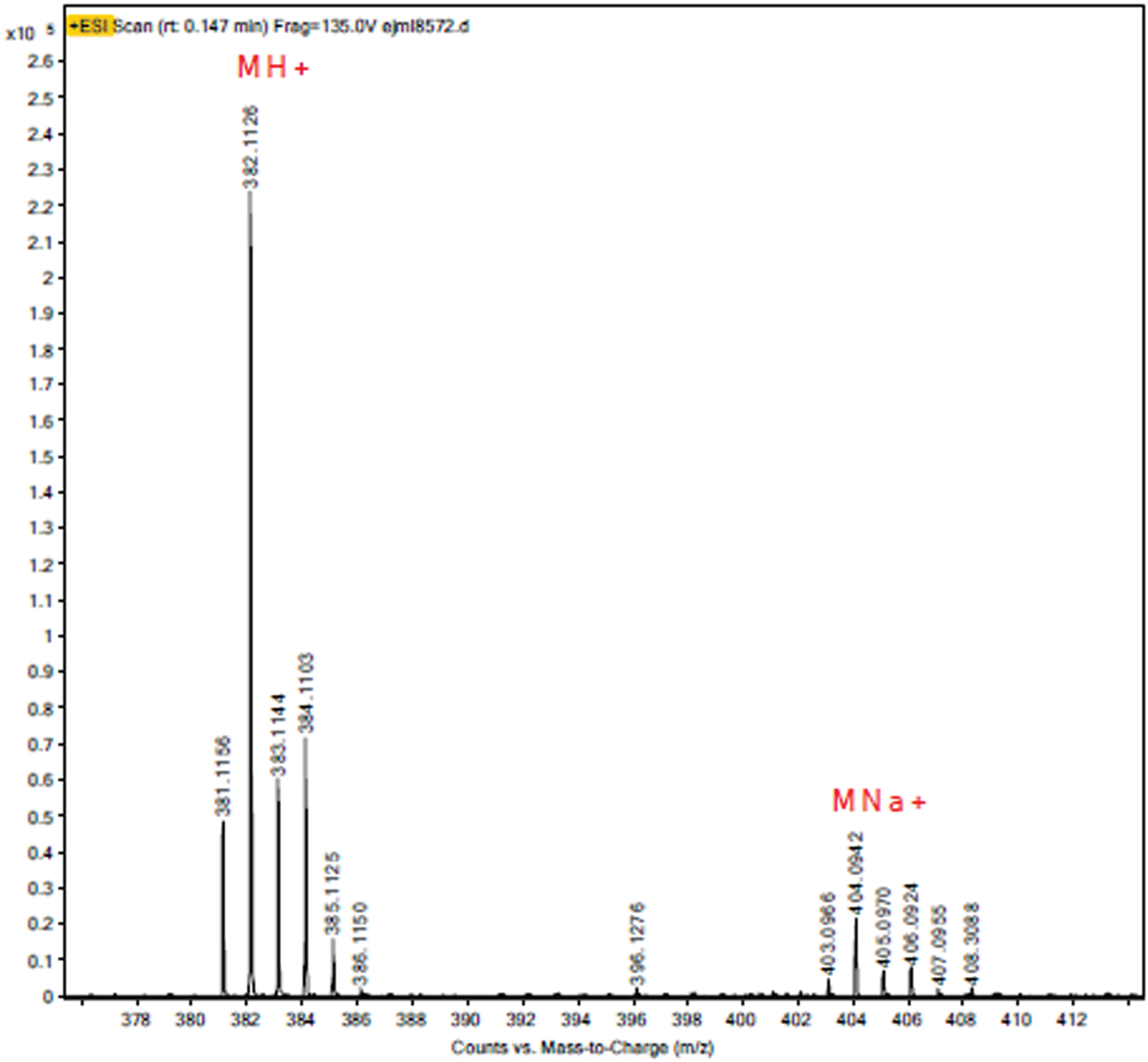

Supplementary Figure 1: Chemical characterization of AN7973. (a) ^{1}H NMR results for AN7973. (b) ^{13}C NMR results for AN7973. (c) High resolution mass spectral (HRMS) data supporting the molecular weight identity of AN7973. Spectra from one of three compound batches tested with similar results are shown.

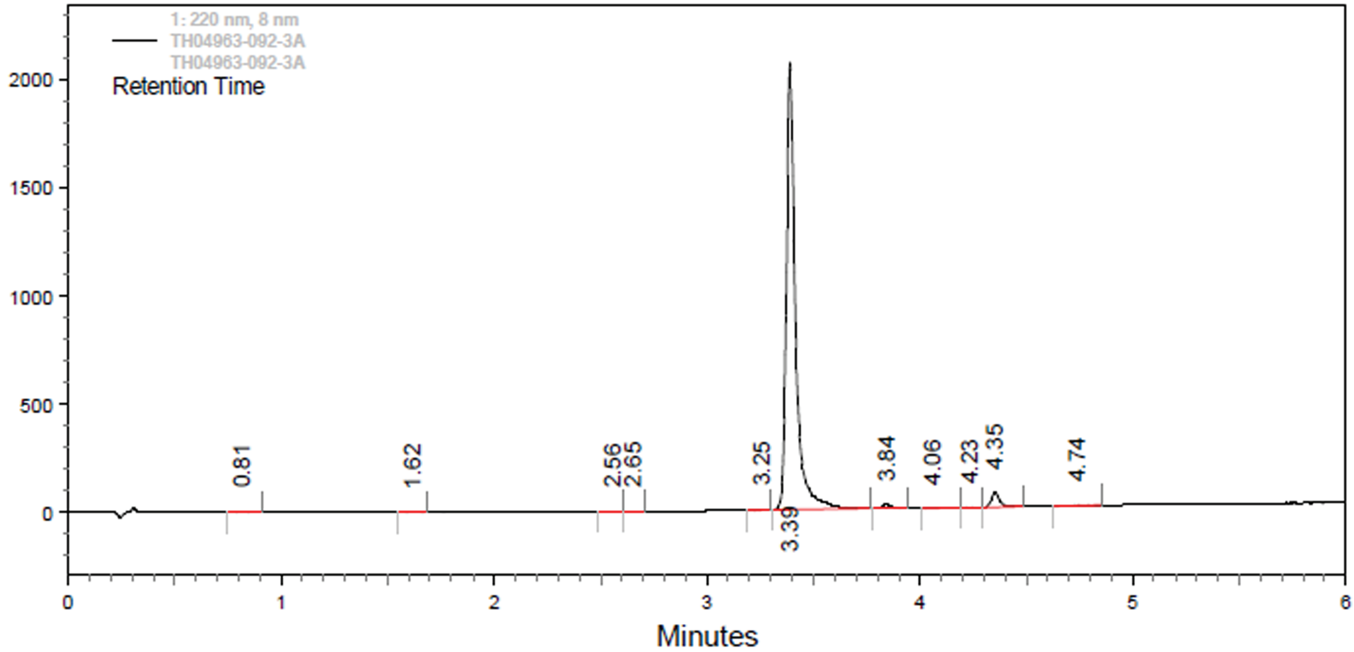

1: 220 nm, 8 nm

Retention Time	*Height*	*Area*	*Area Percent*
0.81	4884	16223	0.25
1.62	1886	5098	0.08
2.56	1421	3606	0.06
2.65	1733	4040	0.06
3.25	3349	8710	0.13
3.39	2042637	6189791	95.39
3.84	19329	57956	0.89
4.06	1569	6477	0.10
4.23	1475	3847	0.06
4.35	64139	185631	2.86
4.74	1711	7613	0.12

Supplementary Figure 2: AN7973 purity. High performance liquid chromatography (HPLC) was used to determine the purity of AN7973. Instrument and column: HPLC-01 Venusil MP C18 5μm 4.6×50 mm. A representative chromatogram and peak area percentages are shown.

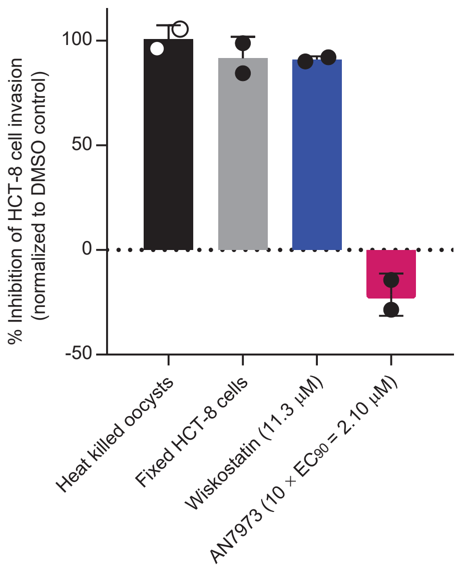

Supplementary Figure 3: Effect of AN7973 on HCT-8 cell invasion by *C. parvum*. *C. parvum* Bunch Grass Farms Iowa strain oocysts were allowed to invade HCT-8 cell monolayers for three hours in the presence of compounds at the indicated concentrations, after which cell monolayers were washed and labeled for immunofluorescence using FITC-conjugated *Vicia villosa* lectin, and parasite vacuoles were quantified using automated microscopy. Heat killed parasites, the ability of viable parasites to invade fixed HCT-8 cell monolayers, and the ability of the known *C. parvum* invasion inhibitor wiskostatin were used as controls (i.e. successful removal of non-invaded parasites by washing). Data were normalized to results using DMSO-treated parasites (mean and SD, n=2 biological replicates).

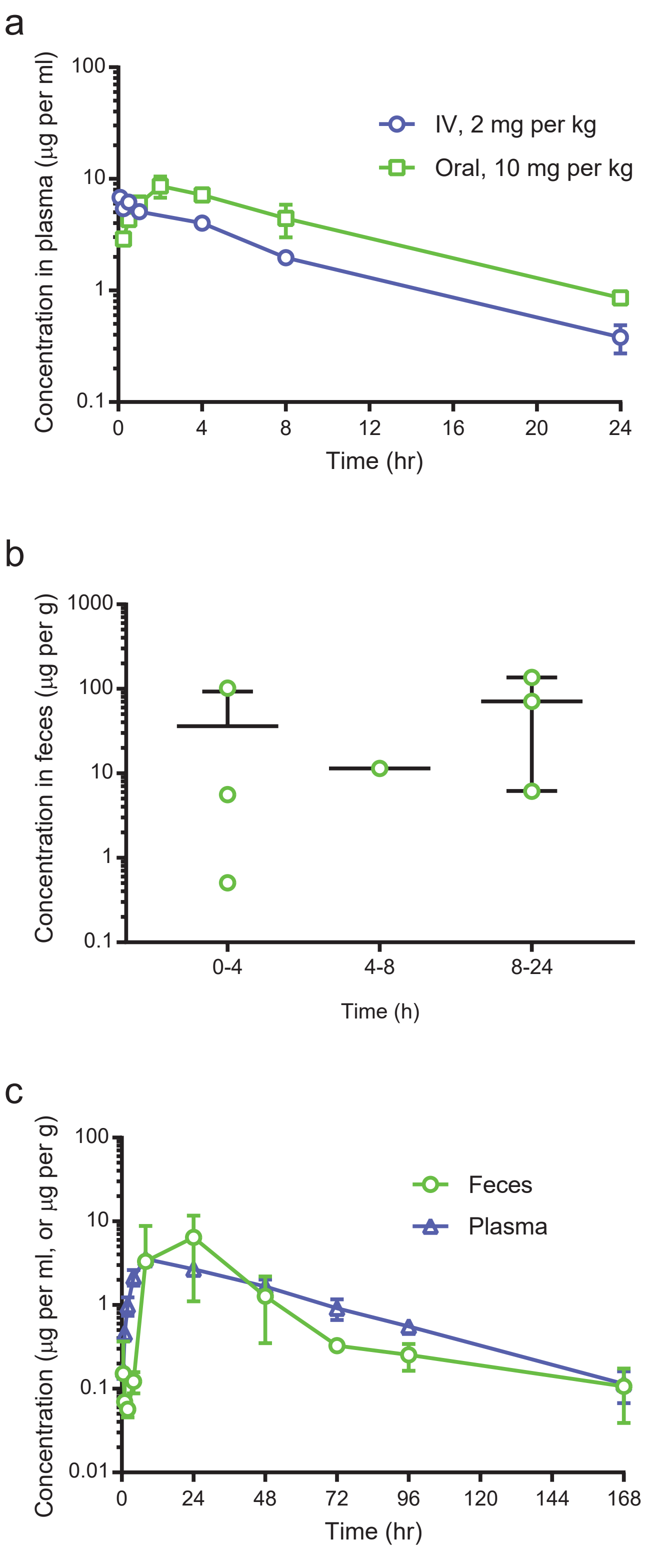

Supplementary Figure 4: Mouse and neonatal calf PK characteristics of AN7973. (a) Concentrations of AN7973 in plasma following administration of a single intravenous (IV) or oral dose to female CD-1 mice. Data are the mean and SD (n=3 mice per group). (b) Concentrations of AN7973 in feces measured at the indicated time intervals following oral administration of a single 10 mg per kg oral dose. Data points show values measured from individual animals (n=3 mice, except for the 4-8 h time interval where n=1 due to absent samples). Lines show the mean and SD (note that the 0-4 hour time interval lower error bar is not shown, because it would extend off the plot). (c) Concentrations of AN7973 in plasma (µg per mL) or feces (µg per g) of neonatal calves following a single 5 mg per kg oral dose administered as a suspension. Data are the mean and SD (n=3 calves per group).

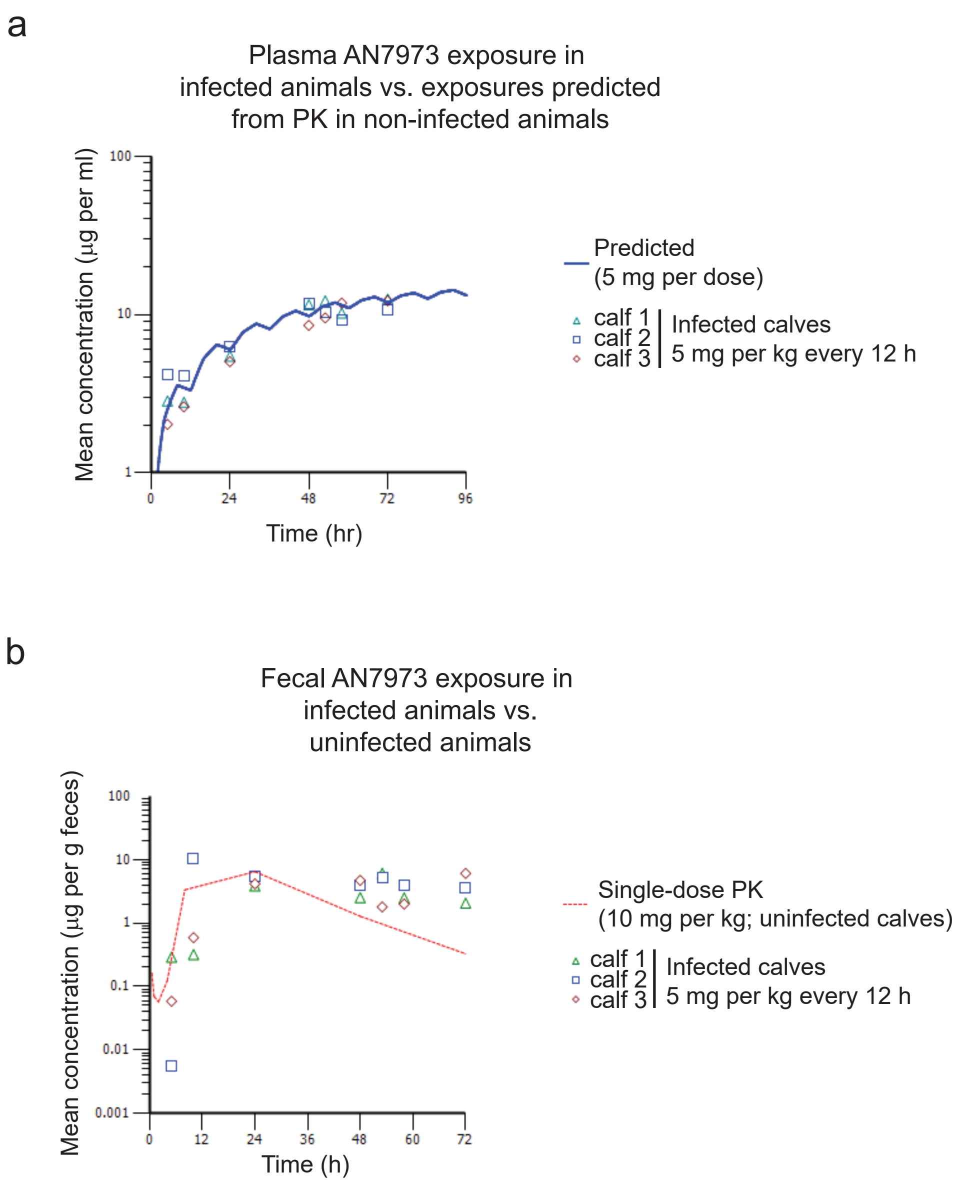

Supplementary Figure 5: Plasma and fecal PK measurements in infected animals vs. uninfected animals. (a) Predicted and measured plasma exposure for infected and uninfected animals administered 5 mg per kg AN7973 orally. Symbols show measurements for individual *C. parvum* infected animals at the indicated times (n=3 calves), and the blue line shows the predicted mean plasma concentration based on the measurements done in uninfected calves that are shown in Supplementary Figure 2c (n=3 calves). (b) Measured fecal exposure for infected calves administered 5 mg per kg AN7973 orally every 12 h (individual data points; n=3 calves), or uninfected calves administered a single 10 mg per kg oral dose (red line; mean, n=3 calves).
