# Supplementary material for: Identification of a potent benzoxaborole drug candidate for treating cryptosporidiosis
Source: Nat Commun. 2019 Jun 27;10:2816. doi: 10.1038/s41467-019-10687-y (PMC6597546; doi:10.1038/s41467-019-10687-y)
Supplement: Supplementary file 2 — Reporting Summary [file 41467_2019_10687_MOESM2_ESM.pdf]

nature research

Corresponding author(s): Christopher D. Huston

Reporting Summary

Nature Research wishes to improve the reproducibility of the work that we publish. This form provides structure for consistency and transparency in reporting. For further information on Nature Research policies, see Authors & Referees and the Editorial Policy Checklist.

Statistical parameters

When statistical analyses are reported, confirm that the following items are present in the relevant location (e.g. figure legend, table legend, main text, or Methods section).

n/a	Confirmed	
☐	☒	The exact sample size (*n*) for each experimental group/condition, given as a discrete number and unit of measurement
☐	☒	An indication of whether measurements were taken from distinct samples or whether the same sample was measured repeatedly
☐	☒	The statistical test(s) used AND whether they are one- or two-sided<br>*Only common tests should be described solely by name; describe more complex techniques in the Methods section.*
☒	☐	A description of all covariates tested
☐	☒	A description of any assumptions or corrections, such as tests of normality and adjustment for multiple comparisons
☐	☒	A full description of the statistics including central tendency (e.g. means) or other basic estimates (e.g. regression coefficient) AND variation (e.g. standard deviation) or associated estimates of uncertainty (e.g. confidence intervals)
☒	☐	For null hypothesis testing, the test statistic (e.g. *F*, *t*, *r*) with confidence intervals, effect sizes, degrees of freedom and *P* value noted<br>*Give P values as exact values whenever suitable.*
☒	☐	For Bayesian analysis, information on the choice of priors and Markov chain Monte Carlo settings
☒	☐	For hierarchical and complex designs, identification of the appropriate level for tests and full reporting of outcomes
☒	☐	Estimates of effect sizes (e.g. Cohen's *d*, Pearson's *r*), indicating how they were calculated
☐	☒	Clearly defined error bars<br>*State explicitly what error bars represent (e.g. SD, SE, CI)*

Our web collection on statistics for biologists may be useful.

Software and code

Policy information about availability of computer code

Data collection	NIS-Elements Advanced Research software (Nikon, version 4.30.01).
Data analysis	GraphPad Prism version 6.01, ChemBioDraw Ultra (PerkinElmer, version 14.0.0.117), ImageJ platform (National Institutes of Health, version 1.52a) with custom macros, Adobe Illustrator version CS5, GE InCell Developer (version 1.9), Phoenix version 6.4 (Pharsight Corporation, Mountain View, CA, USA), CytoSoft data acquisition software (version 5.3; Guava Technologies, Inc.).

For manuscripts utilizing custom algorithms or software that are central to the research but not yet described in published literature, software must be made available to editors/reviewers upon request. We strongly encourage code deposition in a community repository (e.g. GitHub). See the Nature Research guidelines for submitting code & software for further information.

Data

Policy information about availability of data

All manuscripts must include a data availability statement. This statement should provide the following information, where applicable:

- Accession codes, unique identifiers, or web links for publicly available datasets
- A list of figures that have associated raw data
- A description of any restrictions on data availability

All data generated and/or analyzed during the study of AN7973 are available from the corresponding author, CDH, upon request. The source data for Figures 2, 3, 4, 5 and Supplemental Figure 1 are provided as a Source Data File.

Field-specific reporting

Please select the best fit for your research. If you are not sure, read the appropriate sections before making your selection.

[X] Life sciences [ ] Behavioural & social sciences [ ] Ecological, evolutionary & environmental sciences

For a reference copy of the document with all sections, see nature.com/authors/policies/ReportingSummary-flat.pdf

Life sciences study design

All studies must disclose on these points even when the disclosure is negative.

Sample size	Using Statistical Solutions' LCC's software, a power calculation determined that four mice per experimental group would provide a power of 80% to detect a reduction in parasite shedding equivalent to that observed with the positive control, paromomycin at 2000 mg/kg once daily for four days compared to the vehicle control.
Data exclusions	No data was not excluded from analyses.
Replication	In vitro data were complied from 2 to 4 biological replicates with 4 technical replicates per experiment in most cases.<br>Based on animal care guidelines by the University of Vermont Institutional Animal Care and Use Committee, efficacy studies in mice and calves could not be replicated.
Randomization	Prior to infection, mice and calves were randomly assigned to different groups with arbitrary labels for compound dosing.
Blinding	To avoid any bias, microscopy image acquisition and analysis were all automated.<br>To determine the efficacy of compounds in animals, investigators were blinded with labels for animals and for qPCR analysis of fecal samples.

Reporting for specific materials, systems and methods

Materials & experimental systems

n/a	Involved in the study
[X]	[ ] Unique biological materials
[ ]	[X] Antibodies
[ ]	[X] Eukaryotic cell lines
[X]	[ ] Palaeontology
[ ]	[X] Animals and other organisms
[X]	[ ] Human research participants

Methods

n/a	Involved in the study
[X]	[ ] ChIP-seq
[ ]	[X] Flow cytometry
[X]	[ ] MRI-based neuroimaging

Antibodies

Antibodies used	Click-iT® EdU assay kit (Thermo Fisher Scientific, Catalog# C10340) was used as per the manufacturer's instructions. Fluorescein-labeled Vicia villosa lectin (Vector Laboratories, Catalog# FL-1231) was used at 1.33 µg/mL, biotinylated Vicia villosa lectin (Vector Laboratories, Catalog# B-1235) was used at 0.5 µg/mL, Hoechst 33258 (AnaSpec, Catalog# AS-83219) was used at 0.09 mM, and Cy3-streptavidin (Jackson ImunoResearch, Catalog# 016-160-084) at 0.5 µg/mL.. For DMC1 staining monoclonal antibody clone 1HG10G7 (GenScript) neat culture supernatant was used with a secondary Alexa Fluor 568 goat anti-mouse IgG antibody (Invitrogen, catalog# A-11004) at 1:500 dilution (4 µg/mL). Fluorescein isothiocyanate-conjugated mouse anti-Cryptosporidium antibody (Bio-Rad) was used at 0.25 µg / fecal sample.

Validation	Click-iT® EdU assay kit (Thermo Fisher Scientific, Cat# C10340) has been validated on mammalian cells (Salic A, et al. 2008 PNAS). The kit was further validated for mammalian cells and Cryptosporidium parvum by Jumani RS et al. 2019 Nat Commun, and negative (including no EdU) and positive controls (including MMV000760) were used while running each assay. Assays involving Vicia villosa lectin (VVL) (Vector Laboratories) included relevant controls for assays including uninfected staining control and VVL has been validated for immunofluorescence staining of Cryptosporidium parasites by Bessoff K, et al. 2014 AAC, Jumani RS, et. al. 2018 AAC, Jumani RS, et al. 2019 Nat Commun among others. DMC1 antibody validation has been previously published by Jumani RS, et al. 2019 Nat Commun, and MMV665917 (positive control) was included along with other relevant controls in every experiment.

Eukaryotic cell lines

Policy information about cell lines

Cell line source(s)	HCT-8 [HRT-18] were purchased from ATCC (Catalog# CCL-244), Madin-Darby canine kidney (MDCK) type 2 also purchased from ATCC (Catalog# CRL-2936)
Authentication	None of the cell lines used were authenticated.
Mycoplasma contamination	These cell lines were not tested for contamination with mycoplasma.
Commonly misidentified lines (See ICLAC register)	No misidentified cell lines were used in this study.

Animals and other organisms

Policy information about studies involving animals; ARRIVE guidelines recommended for reporting animal research

Laboratory animals	NOD.Cg-Prkdcscid Il2rgtm1Wjl/SzJ strain # 005557 "NOD Scid gamma (NSG)". All NSG mouse studies were performed in compliance with animal care guidelines and were approved by the University of Vermont Institutional Animal Care and Use Committee. Male NSG mice were purchased from The Jackson Laboratories (Bar Harbor, ME) at the age of three week old (± 3 days) and allowed to acclimatize for one week before start of the study. In compliance with animal care guidelines WuXi Biologics (Wuxi, China) performed single-dose oral and intravenous murine PK studies in female CD-1 mice. All IFN-γ knockout mouse studies were performed in compliance with animal care guidelines and with approval by the Explora BioLabs (San Diego, CA) Institutional Animal Care and Use Committee. Female C57BL/6 IFN-γ-/- mice with normal flora at the age of four weeks were purchased from The Jackson Laboratory (Bar Harbor, ME). The mice were acclimated for 3 days before the experiments.
Wild animals	All calf efficacy studies were conducted in compliance with the USDA-APHIS "Blue Book", available at www.aphis.usda.gov/animal-welfare and were approved by the University of Vermont Institutional animal Care and Use Committee. From Green Mountain Dairy (Sheldon, VT) Holstein bull calves were acquired at birth and within two hours of birth were given synthetic colostrum with 200 g of IgG (Land O'Lakes, Ardent Hills, MO) and bovine coronavirus and Escherichia coli antibodies (First Defense Bolus, Immuncell Corporation, Portland, ME). Calves were then transported to UVM, group-housed initially, and infected at 24-48 hours of birth.
Field-collected samples	This study did not involve any samples collected from the field.

Flow Cytometry

Plots

Confirm that:

- [ ] The axis labels state the marker and fluorochrome used (e.g. CD4-FITC).
- [ ] The axis scales are clearly visible. Include numbers along axes only for bottom left plot of group (a 'group' is an analysis of identical markers).
- [ ] All plots are contour plots with outliers or pseudocolor plots.
- [ ] A numerical value for number of cells or percentage (with statistics) is provided.

Methodology

Sample preparation	*Describe the sample preparation, detailing the biological source of the cells and any tissue processing steps used.*
Instrument	Guava EasyCyte flow cytometer.
Software	CytoSoft<br>404 data acquisition software (version 5.3; Guava Technologies, Inc.).
Cell population abundance	*Describe the abundance of the relevant cell populations within post-sort fractions, providing details on the purity of the samples and how it was determined.*

Gating strategy

Describe the gating strategy used for all relevant experiments, specifying the preliminary FSC/SSC gates of the starting cell population, indicating where boundaries between "positive" and "negative" staining cell populations are defined.

☐ Tick this box to confirm that a figure exemplifying the gating strategy is provided in the Supplementary Information.
